# Recruitment and retention in young adult health research: Motivations and barriers

**DOI:** 10.1017/cts.2025.10182

**Published:** 2025-10-22

**Authors:** Ruixiao Wang, Julie Schweitzer, Gloria Zavala Perez, Silvia C. Molina, Theresa H.M. Keegan

**Affiliations:** 1 Clinical and Translational Science Center, University of California Davis, Sacramento, CA, USA; 2 Department of Psychiatry and Behavioral Sciences and MIND Institute, University of California Davis, Sacramento, CA, USA; 3 Division of Hematology and Oncology, Center for Oncology Hematology Outcomes Research and Training (COHORT), University of California, Davis Comprehensive Cancer Center, Sacramento, CA, USA

**Keywords:** Adolescent, young adult, recruitment, retention, health studies

## Abstract

**Objective:**

To provide insights into the motivations, challenges, and preferred methods of contact that influence the recruitment and retention of young adults (YAs) in health research.

**Methods:**

We designed, collected, and analyzed two surveys targeting YAs aged 18–39 years through the Amazon MTurk platform, to assess factors influencing recruitment and retention in health studies. The recruitment survey (*n* = 477) examined initial engagement motivations, while the retention survey (*n* = 473) explored factors that sustain long-term participation. Descriptive analyses were stratified by age group and sex.

**Results:**

The recruitment survey indicated that 88% of YAs were willing to participate in health studies, with a preference for online formats (78%). Social media, particularly Facebook (53%), was endorsed as the most common platform for discovering research opportunities. Monetary incentives were reported as the top motivator across all age groups, especially for those aged 35–39 years, with gift cards endorsed as the most appealing to participants aged 18–34. Retention survey results indicated that email (100%) was the most preferred method for maintaining engagement, followed by text messages (78.9%) and social media (62.2%). Text messages (65%), regular updates (56%) and sharing of study results (54%) were identified as key factors for maintaining participant engagement.

**Conclusion:**

Our findings identify that YA participation is driven by a mix of altruistic motivations, such as contributing to the community and research, and personal motivations, including personal health benefits and financial incentives, emphasizing the need for strategies that address both aspects of recruitment and retention motivations.

## Introduction

Young adults (YAs) face unique challenges in health outcomes, yet their participation in health research remains limited. In the United States, YAs are commonly defined as individuals aged 18 to 39 in oncology research and practice [1–3]. However, definitions vary across disciplines. Psychological research recognizes individuals as emerging adults (18–25) and YAs (25–35) [4,5], which reflects emerging evidence of prolonged brain development and changing social patterns, including delayed traditional adult milestones [4]. Each developmental stage includes different levels of independence, social obligations, and healthcare needs. Emerging adults frequently navigate key transitions, such as advancing in education, securing employment, and adopting independent healthcare behaviors, yet many still rely on parental guidance [6]. In contrast, YAs demonstrate greater autonomy, independently managing their finances, schedules, and healthcare decisions [6,7]. These variations influence how individuals access information, assess health benefits and risks, and engage with research activities. Therefore, tailored strategies to promote research participation must be tailored to account for these developmental and social factors across the YA population.

Large-scale, longitudinal epidemiologic projects focusing on participants in this YA age range, such as the Coronary Artery Risk Development in Young Adults Study (CARDIA) and the Environmental Influences on Children’s Health Outcomes (ECHO) that ranges from preconception to emerging adulthood, point to the recognition of the importance of increasing our understanding of development during these critical periods and require strong enrollment and retention to be successful [8,9]. Despite the growing attention to this population, improving health outcomes for this population has been challenging due to their low initial participation in research studies. Recruitment is crucial, as it ensures a representative sample. Additionally, retention is important, as even a loss of less than 5% or participants may result in biased results, whereas a loss of 20% or more significantly affects the internal validity of a study [10].

Each year, approximately 90,000 adolescents and YAs are diagnosed with cancer in the U.S., with breast cancer, thyroid cancer, melanoma, colorectal cancer, cervical cancer, testicular cancer, lymphoma, sarcoma and leukemia being the most commonly diagnosed diseases [11–13]. Cancer ranks as the foremost disease-related cause of death among YAs in the U.S., consistently appearing as one of the leading causes across this age range. Specifically, cancer represents the highest-ranking disease-related cause of death and the fourth leading cause of death overall among individuals aged 15–34, rising to the third leading cause of death overall among those aged 35–44 [14]. YA cancer survivors have been observed to have worse survival or less survival improvements compared to younger children and older adults for a number of cancers, prompting national initiatives to improved outcomes in the age group, including increasing participation in clinical trials [11,15]. YA patients with cancer have substantially lower enrollment rates in clinical trials than children with cancer [16], leading to a gap in optimizing care strategies and treatments tailored for YAs in oncology. In addition, participation in cancer survivorship studies that can obtain important information on care quality and physical and mental health outcomes has been found to be lower among YAs than older adults, likely due to this population being mobile and difficult to contact/follow due to moves related to education, employment, marriage or other life changes [15–17]. In our previous comprehensive review, we identified existing recruitment and retention strategies for adolescent and YA cancer survivors, revealing a significant reliance on digital and social media outreach, which offers higher engagement rates compared to traditional methods. However, critical gaps remain in developing tailored approaches that address YAs’ distinct lifestyle and communication preferences [18].

Mental health disorders are significant contributors to suicide among YAs. Suicide is the second leading cause of death for individuals aged 25–34, and the fourth for those aged 35–44 [14]. Approximately 5% of adults aged 18 and older regularly experience feelings of depression; depressive disorders account for 15 million physician office visits annually, representing about 11% of all physician office visits in the U.S. [19]. Attention-deficit/hyperactivity disorder (ADHD), one of the most prevalent childhood and adult psychiatric disorders, was long considered a childhood disorder, but is now recognized to continue into adulthood with, many newly diagnosed with ADHD when YAs [20–23]. Maintaining research engagement with youth diagnosed with ADHD or other disorders assessed in childhood (e.g., autism) can be challenging when they are YAs and their care is no longer managed by their parents. Research on emerging to YA developmental stage is critical as YAs strive for independence and self-identity while beginning to engage in behaviors that pose significant health risks, such as substance use and high-risk sexual activities [24]. Moreover, challenges, such as managing education, employment, and social functioning, are compounded by increasing rates of anxiety, depression, and ADHD [25]. Developmental factors often hinder effective communication between YAs and healthcare providers, creating barriers to preventive care [26]. Longitudinal health studies are vital for understanding the evolution of health behaviors and outcomes over time [27], yet recruitment and retention of YAs in these studies remain challenging, particularly in mental health research, where targeted strategies are still underdeveloped [10,28].

Understanding the factors that influence the recruitment and retention of YAs in longitudinal studies is essential for enhancing patient outcomes and advancing research. Our study aims to provide insights into the motivations, challenges, and preferred ways of contact for the YA population in the context of health studies by assessing the barriers and facilitators identified through surveys administered to the general YA population. We present our survey results for YAs stratified by sex and age group given differences in participation found in prior studies [15,17] and to provide more granular data for investigators conducting YA research.

## Method

### Survey design

Two surveys were conducted – a recruitment survey and a retention survey. The surveys were developed based on a review of published literature identifying barriers and facilitators to research participation among YAs, with a focus on digital communication, incentive strategies, and engagement preferences [18]. The wording of the questions was reviewed and edited based on feedback from an adolescent and YA health research advisory board before they were launched. This advisory board is composed of individuals aged 13 to 39 years, representing diverse racial, ethnic, and educational backgrounds. The advisory board input ensured the language and structure of survey items were appropriate, culturally sensitive, and understandable for our audience. Both surveys collected demographic information on age, sex, race and ethnicity, level of education, and type of employment. Survey questions were designed to be multiple choice, with some allowing participants to select multiple options and provide text entries. In the recruitment survey, questions included whether respondents would consider participating in a health study or clinical trial, reasons for participating or withdrawing from studies, their attitudes toward online and in-person research participation, whether they had previously heard about opportunities to participate in health studies or clinical trials, the platforms through which they learned about these opportunities, and best ways of sending out information about studies (Supplemental Table 1). In the retention survey, questions included best ways and platforms to keep in contact with participants, best ways to motivate participants to stay in the studies, attitude toward learning the results of the study, as well as ways to share results with participants (Supplemental Table 2).

### Data collection

Amazon Mechanical Turk (MTurk, www.mturk.com) was used as a platform to collect survey data from August 2022 to May 2023. MTurk is an increasingly utilized online marketplace operated by Amazon.com that allows researchers to access a large and diverse pool of participants at lower costs. It has similar demographic distributions with other survey services and attracts more young individuals compared to other survey platforms [29]. Registered users must be at least 18 years old, meet certain criteria determined by the requesters, and need a computing device connected to the Internet to complete tasks and collect payments [30,31].

### Participants

Registered MTurk users within the YA age range of 18–39 years old were included. The exclusion criteria were 1) respondents older than 39 years old; and 2) incomplete survey responses. For the recruitment survey, 636 responses were initially received. After excluding incomplete responses (*N* = 125) and participants outside the age range of 18–39 (*N* = 34), the final number of responses was 477. Similarly, the retention survey initially recruited 739 participants, after excluding incomplete responses (*N* = 201) and those outside the age range of 15–39 (*N* = 65), the final number of responses was 473. Each participant completed surveys posted on Qualtrics and received $2.00 for their time completing the recruitment survey and $1.50 for their time completing the retention survey. Qualtrics recorded de-identified individual responses. The survey was conducted as a quality improvement project. The UC Davis IRB reviewed the project retrospectively and determined the survey research to be exempt.

### Statistical analyses

Descriptive analyses (N sizes and percentages) were used to describe the characteristics of participants completing the recruitment and retention survey. Due to the sex differences in enrollment in trials found in existing literature [32] and the wide age range of our participants, the survey question results were stratified and presented by sex and age (N sizes and percentages). With the exception of the youngest age group, where the number of participants was lowest, we considered five-year age groups (18–24, 25–29, 30–34, 35–39), as done in prior studies [33,34]. Data cleaning and recoding of text responses were performed using SAS 9.4, and figures were generated using Microsoft Excel and R.

## Results

### Participant characteristics

Among the recruitment survey participants, most were aged 35–39 years (50.4%), followed by those aged 30–34 years (37.2%), 25–29 years (9.7%), and 18–24 years (2.7%) (Table 1). Most participants were male (62.5%) and of non-Hispanic White (64.0%) or Asian (20.6%) race/ethnicity. Nearly half of the respondents had a bachelor’s degree (49.7%) and the majority of participants were employed full-time (82.0%).

**Table 1.** Characteristics of young adult participants in the recruitment and retention surveys

Characteristic	Recruitment Survey *N* = 477 n (%) †	Retention Survey *N* = 473 n (%) †
Age (years)		
18–24	13 (2.7)	7 (1.6)
25–29	46 (9.7)	37(8.3)
30–34	177 (37.2)	144 (32.2)
35–39	240 (50.4)	259 (57.9)
Sex		
Female	176 (37.5)	154 (34.5)
Male	294 (62.5)	293 (65.5)
Race/ethnicity		
Non-Hispanic White	301 (64.0)	295 (66.0)
Non-Hispanic Black	27 (5.7)	25 (5.6)
Asian	97 (20.6)	86 (19.2)
Hispanic	24 (5.1)	24 (5.4)
Others*	21 (4.5)	17 (3.8)
Educational level		
High School	68 (14.5)	68 (15.2)
Some College	117 (25.0)	110 (24.6)
Bachelor’s Degree	233 (49.7)	220 (49.2)
Master’s Degree	51 (10.9)	49 (11.0)
Employment Status		
Full-time	379 (82.0)	379 (84.8)
Part-time	61 (13.2)	53 (11.9)
Unemployed	22 (4.8)	15 (3.4)

*Other races include American Indian/Alaska Native and Hawaiian/Pacific Islander. † Column percentages may not sum to 100% due to rounding.

Participants completing the retention survey were primarily aged 35–39 (57.9%) and 30–34 years (32.2%) (Table 1). As in the recruitment survey, most participants were male (65.5%) were of non-Hispanic White (66.0%) or Asian (19.2%) race/ethnicity, had a bachelor’s degree (49.2%), and had full-time employment (84.8%).

### Recruitment survey findings

Most respondents (88%) were willing to participate in a health study or clinical trial, while 8% reported that they may consider participating and 5% were reluctant to participate. The majority (78%) indicated their interest in joining a study would differ depending on whether the study was online or in person. Seventy percent of the respondents have heard about opportunities to participate in heath studies or clinical trials in the past, whereas 26% have not, and the remaining 4% answered “maybe.” In terms of the social media platforms where people have heard about these opportunities, Facebook was the most cited (53%), followed by Reddit (33%), YouTube (27%), and Twitter (22%). Less common platforms included LinkedIn (9%), Discord (3%), Snapchat (2%), and Twitch (2%). Regarding non-social media sources, participants most commonly heard about health studies through letters/brochures (43%), emails (39%), online ads (37%), and television (TV, 27%). Least mentioned were phone calls (5%), radio (8%), and school announcements (8%).

**Figure 1.**
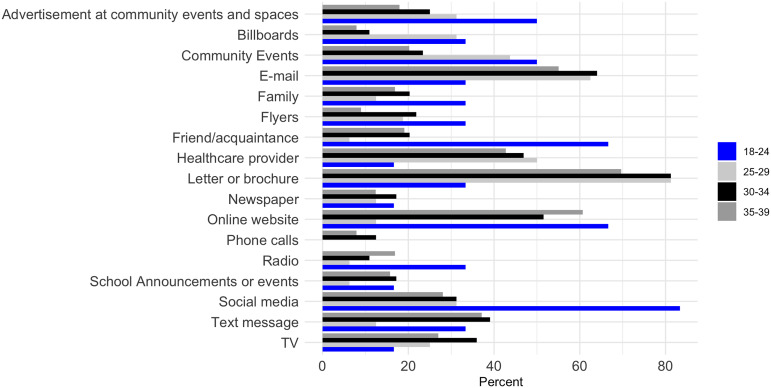
Preferred ways to send out information to female young adults about health studies.

**Figure 2.**
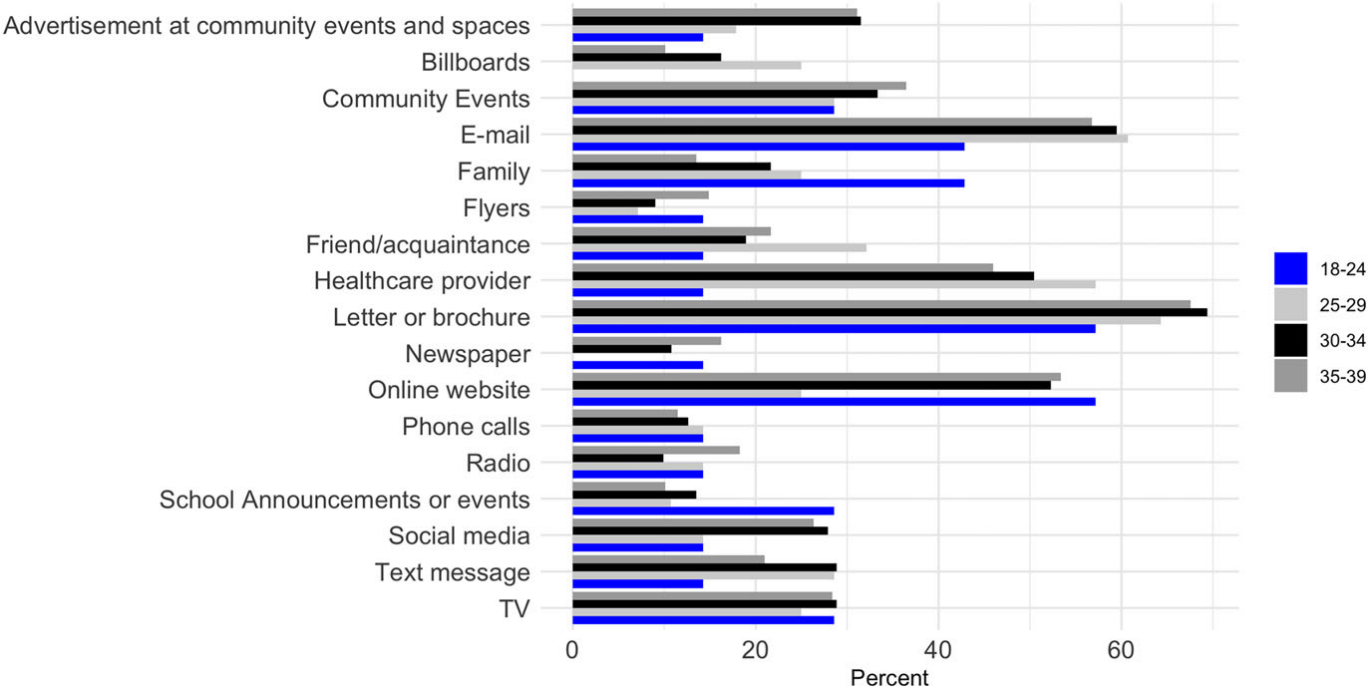
Preferred ways to send out information to male young adults about health studies.

For females aged 18–24 years, social media (83%), friend and acquaintance recommendation (67%), and online websites (67%) were the most preferred ways to receive information (Figure 1). Females aged >25 favored emails, letters or brochures, and healthcare provider recommendations. For males aged 18–24, online websites (57%) and letters or brochures (57%) were the most preferred methods; while among older males, emails and letters or brochures were identified as the preferred ways of sending out information (Figure 2).

**Figure 3.**
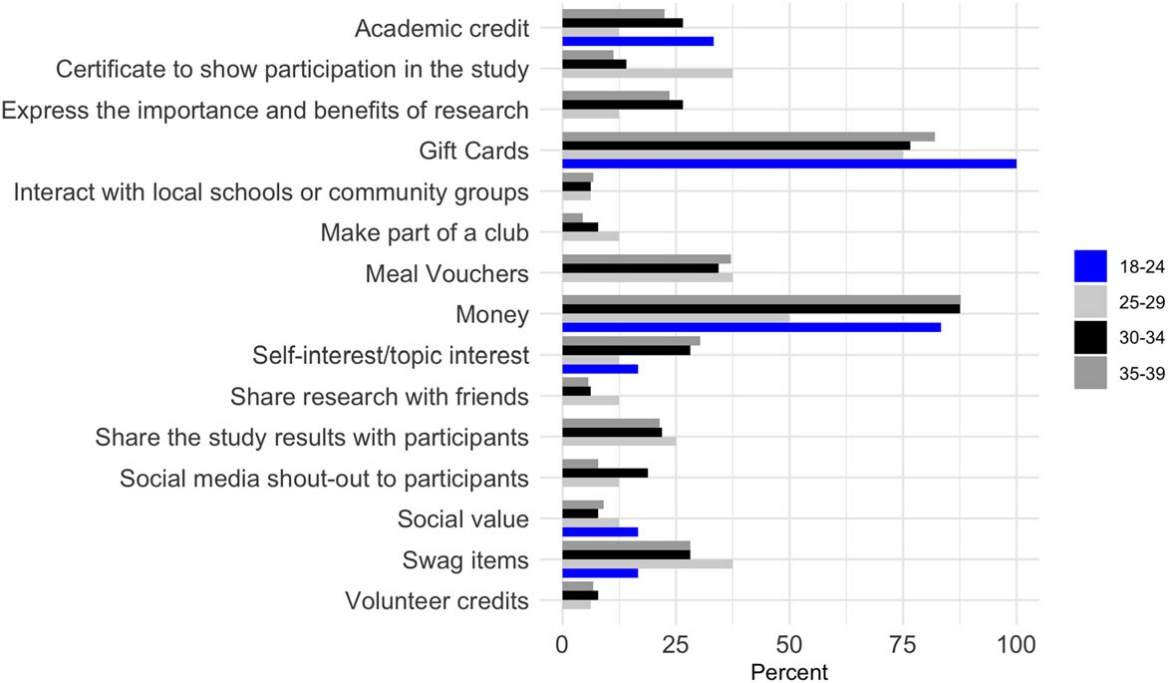
Motivation for female young adult participation in research studies by age group.

**Figure 4.**
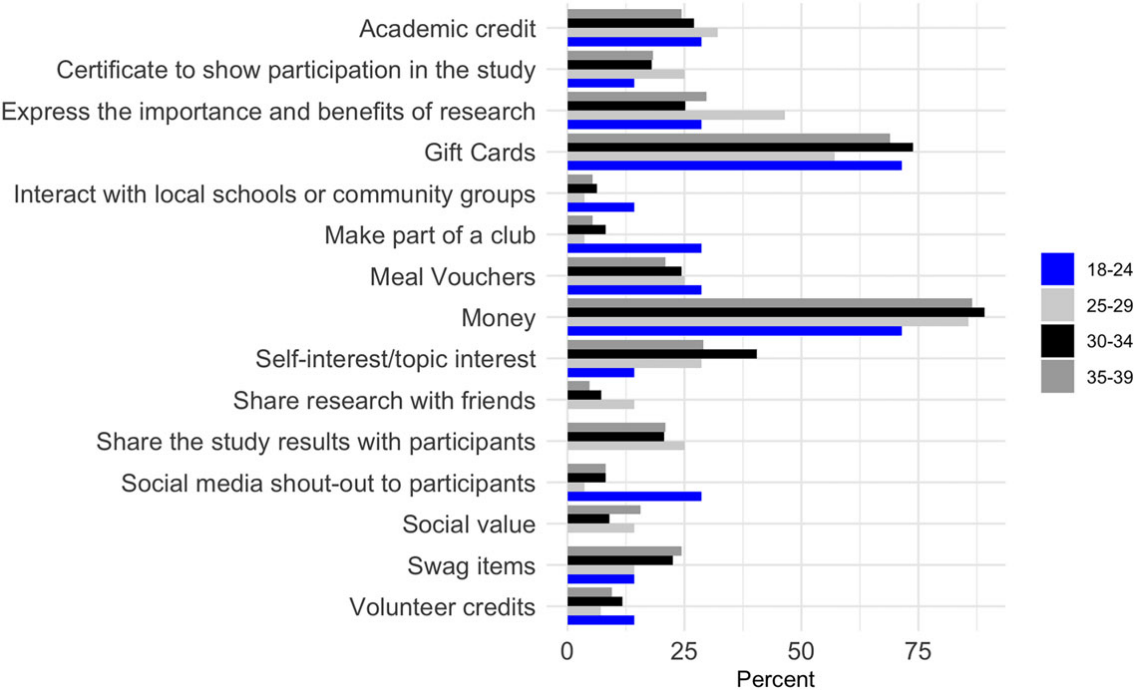
Motivation for male young adult participation in research studies by age group.

The primary motivation for participating in research studies among females across all age groups was financial compensation, with the strongest interest seen in the 35–39 age group (Figure 3). Gift cards were also a motivator, particularly for younger females aged 18–24. Academic credit and certificates showing participation were more appealing to the 18–24 and 25–29 age groups, respectively. Expressing the importance and benefits of research resonated strongly with the 30–34 age group, as well as meal vouchers, self-interest or topic interest. Results were similar for male participants, with money being a key motivator, especially for those aged 35–39 years (Figure 4). Gift cards were also highly attractive, particularly to the 18–24 and 30–34 age groups. The 25–29 age group showed substantial interest in receiving academic credit and certificates for participation.

The most significant barriers to participation in health studies or clinical trials, as reported by respondents across all age groups, were potential adverse side effects, inadequate incentives, time constraints, and the burden of travel distance (Table 2). Among female participants aged 18–24 years, other primary concerns included uncertainty about study procedures (50.0%), study takes too much time (50.0%), and other time commitments (50.5%). For females aged >25, cost and lack of flexible scheduling were other leading barriers. For male participants aged 18–24, concerns about sharing personal information (28.6%) and needing to miss work (28.6%) were notable barriers. For older males, other top barriers included taking too much time, and lack of flexible scheduling.

**Table 2.** Factors that would either stop or encourage young adult participation in a health study, by sex and age group (years)

	Female^*^				Male^*^			
	18-24%	25–29%	30–34%	35–39%	18–24%	25–29%	30–34%	35–39%
**Stopping participation**								
Concern or fear about possible side-effects	83.3	62.5	71.9	80.9	42.9	42.9	77.5	65.5
Insufficient incentives	33.3	50.0	56.3	57.3	0.0	42.9	64.0	57.4
Travel distance	33.3	37.5	46.9	47.2	42.9	25.0	44.1	41.2
Takes too much time	50.0	43.8	32.8	46.1	0.0	39.3	51.4	43.2
Lack of flexible scheduling	33.3	37.5	40.6	43.8	0.0	28.6	44.1	36.5
Too expensive	16.7	50.0	31.3	39.3	14.3	17.9	38.7	35.1
Concern or uncertainty about study procedures	50.0	31.3	34.4	37.1	0.0	25.0	29.7	21.6
Other time commitments (school, sports, job)	50.0	18.8	34.4	37.1	14.3	35.7	32.4	32.4
Concern about sharing personal information	16.7	25.0	31.3	34.8	28.6	39.3	37.8	34.5
Would need to miss work	33.3	25.0	18.8	32.6	28.6	28.6	36.9	38.5
Transportation challenges	33.3	18.8	31.3	27.0	0.0	17.9	25.2	22.3
Concern that you will be in the control group and not get treatment	0.0	25.0	25.0	22.5	14.3	14.3	12.6	16.2
Language barriers	0.0	12.5	6.3	13.5	14.3	7.1	10.8	4.7
Social pressure (stigma/worried about what others think)	16.7	6.3	4.7	7.9	0.0	10.7	7.2	3.4
Cultural barriers	0.0	25.0	4.7	5.6	0.0	7.1	1.8	5.4
Do not have the technology (computer, cellphone)	16.7	0.0	1.6	3.4	0.0	3.6	4.5	0.7
Limits your participation in another research study	0.0	0.0	0.0	0.0	0.0	0.0	0.0	0.0
**Encouraging participation**								
Incentive	83.3	56.3	84.4	77.5	0.0	60.7	77.5	66.9
Improving treatment	16.7	62.5	48.4	61.8	28.6	35.7	52.3	50.0
Getting test results	50.0	62.5	50.0	52.8	28.6	32.1	55.0	50.0
Getting study results	50.0	56.3	45.3	49.4	28.6	28.6	40.5	38.5
Friend/family referral	16.7	25.0	29.7	36.0	28.6	57.1	33.3	32.4
Health care provider referral	33.3	12.5	39.1	27.0	0.0	32.1	23.4	16.9
Learning about research	50.0	31.3	21.9	22.5	0.0	17.9	18.0	18.2
Giving back to the community	33.3	18.8	26.6	16.9	0.0	17.9	28.8	20.3
Time commitment of study	33.3	6.3	9.4	12.4	0.0	7.1	11.7	6.8
Self-interest	0.0	6.3	6.3	7.9	14.3	10.7	8.1	10.1

* Column percentages may not sum to 100% due to rounding.

Across all age groups and both sexes, the provision of incentives and access to test results emerged as consistent motivators for participation in health studies (Table 2). Among females aged 18–24, 40% were particularly motivated by the opportunity to learn about research, whereas females in other age groups cited the potential to improve treatment as a key incentive. For males aged 18–24, self-interest was a significant motivator, while males across all age groups indicated that contributing to the improvement of treatment was a primary reason for participation.

### Retention survey

Among the retention survey respondents, 100% of them agreed that email is the best way to keep in contact with people over time, followed by text messages (78.9%) and social media (62.2%). Specifically, Facebook (72%) was the top mentioned social media platform, followed by Instagram (45%), Twitter (45%), Discord (34%), WhatsApp (29%), and Reddit (26%). Eighty-two percent of the respondents reported wanting to learn about the results of the research if they participated in a study, while only 4% were not interested in learning the results, and the remaining 14% were not sure. Among the methods for sharing research results with participants, the most favored method was email (85%), followed by posting results on a website (45%), text messages (38%), and social media (36%). The most favored social media platforms for sharing results included: Facebook (59%), YouTube (36%), Twitter (36%), Reddit (30%), Instagram (29%), Discord (26%), and WhatsApp (21%). Other social media platforms were less commonly endorsed: LinkedIn (14%), TikTok (9%), Snapchat (4%), Pinterest (4%) and Twitch (4%). Sharing the results through meetings with researchers (22%), videos (22%), published papers (19%), newsletters (18%), blogs (18%), and podcasts (9%) were less preferred.

To maintain participant engagement and involvement in a long-term study, sharing study results proved to be a highly effective strategy among females across all age groups. This approach was particularly impactful for those aged 18–24 (66.7%) and 25–29 (77.8%) years (Table 3). Forming a community with other participants was also rated as valuable for study retention, especially for ages 25–29 (77.8%) and 35–39 (58%). Regular check-ins with participants and text messages were particularly valued by older age groups, with check-ins being most effective for ages 30–34 (68.8%) and text messages for ages 30–34 (81.3%) for study retention. For male participants, text messages were endorsed as the most effective method for ages 18–24 (100%) and 25–29 (89.3%), while providing study results was highly valued by ages 25–29 (53.6%) and 35–39 (58.3%) to retain study involvement. Males ages 25 and older endorsed check-ins as methods to maintain involvement.

**Table 3.** Preferred ways to maintain young adult health study participants interested and involved in a longitudinal study and motivate continued participation by sex and age group (years)

	Female^*^				Male^*^			
	18–24%	25–29%	30–34%	35–39%	18–24%	25–29%	30–34%	35–39%
**Maintaining interest and involvement in a longitudinal study**								
Foster relationships	33.3	44.4	31.3	31.0	25.0	28.6	37.0	36.9
Check-in	0.0	55.6	68.8	58.0	0.0	57.1	55.0	54.8
Birthday cards	0.0	33.3	14.6	15.0	0.0	25.0	13.0	13.1
Memes	0.0	11.1	33.3	21.0	25.0	35.7	25.0	16.1
Text messages	0.0	55.6	81.3	65.0	100.0	89.3	63.0	60.1
Provide information on study results	66.7	77.8	54.2	57.0	0.0	53.6	45.0	58.3
Invite the participant to meet-up	0.0	11.1	29.2	24.0	0.0	17.9	27.0	32.7
Form a community with other participants	33.3	77.8	43.8	58.0	25.0	28.6	43.0	49.4
**Motivating continued participation**								
Academic credit	66.7	22.2	27.1	25.0	0.0	39.3	30.0	25.0
Certificate to show participation	0.0	44.4	18.8	10.0	0.0	25.0	13.0	19.0
Express the importance and benefits of research	0.0	22.2	33.3	25.0	0.0	32.1	30.0	25.6
Gift Cards	66.7	88.9	79.2	85.0	75.0	64.3	74.0	72.0
Interact with local schools or community groups	0.0	0.0	16.7	12.0	0.0	10.7	8.0	6.5
Make part of a club	0.0	11.1	2.1	12.0	0.0	7.1	6.0	10.1
Meal Vouchers	33.3	11.1	37.5	28.0	25.0	35.7	26.0	23.8
Money	100.0	66.7	81.3	87.0	100.0	82.1	89.0	85.7
Self-interest/topic interest	33.3	44.4	31.3	29.0	0.0	32.1	38.0	31.5
Share research with friends	0.0	0.0	4.2	7.0	0.0	7.1	11.0	8.3
Share their test results with participants	66.7	44.4	25.0	29.0	0.0	21.4	23.0	27.4
Share the study results with participants	33.3	44.4	25.0	31.0	0.0	28.6	26.0	36.3
Social media shout-out to participants	0.0	0.0	22.9	10.0	0.0	25.0	10.0	11.9
Social value	0.0	0.0	12.5	12.0	0.0	21.4	9.0	14.9
Swag items	66.7	33.3	50.0	43.0	25.0	21.4	28.0	39.9
Volunteer credits	33.3	11.1	14.6	15.0	0.0	7.1	13.0	11.3

* Column percentages may not sum to 100% due to rounding.

Money was rated as the preferred method to motivate continued participation, with females ages 18–24 highly endorsing it (100%) and ages 25–29 (50%), while gift cards were most effective for ages 25–29 (50%) and 30–34 (55%) years (Table 3). Sharing study and test results, self-interest, and expressing the importance of research were also significant motivators across all age groups. For male participants, money and gift cards were consistently top motivators across all age groups, with money being especially effective for ages 18–24 (80%) and 25–29 (55%). Sharing test results and expressing the importance of research were also noted for males across all age groups.

## Discussion

YAs represent a diverse group experiencing significant life transitions that affect their health behaviors and outcomes. The purpose of this study was to identify the unique factors influencing YA participation in research with the aim of improving recruitment and retention strategies and enhancing the validity and applicability of health research within this population. By focusing on the motivations and barriers specific to YAs, this study provides insights that can help tailor research approaches to meet the needs and preferences of this age group more effectively.

The findings from the recruitment survey highlighted a strong expression of willingness among YAs to engage in health research, with 88% respondents reporting their openness to participation. However, this positive attitude may have been influenced by the format of the study, with a significant portion of the respondents (78%) preferring online studies over in-person ones, reflecting the digital-native characteristics of this cohort, meaning individuals who have grown up in the era of digital technology, are accustomed to interacting frequently with online platforms, and are comfortable engaging with digital communication methods [35]. Awareness of research opportunities was substantial, as 70% had previously heard about studies, primarily through social media platforms. The effectiveness of different communication channels varied by age and sex, indicating the need for targeted outreach strategies. Younger respondents favored dynamic and interactive platforms, such as social media for receiving information, whereas older participants within the YA age range showed a preference for more traditional forms, like emails and direct mail.

Our findings can be categorized into altruistic and personal motivations for participating in studies [36]. Altruistic and personal motivations often coexist and can influence participation decisions in complex ways [36,37]. Some altruistic motivations, which encompass the desire to contribute to society or advance research, were more commonly found among older participants in our study. Specifically, improving treatment was endorsed by 62% of female YAs aged 35–39 and 25–29 and 48% of female YAs aged 30–34, compared to only 17% of female YAs <25. Males showed the same pattern, where more than 50% of YAs ≥30 were encouraged to participate in research to improve treatment. Similarly, giving back to the community was a motivation for nearly 50% of males aged 30–39, compared to 18% of males aged 25–29 and no male YAs <25. Research suggests that as individuals age, personal motivations commonly get replaced by altruistic motivations and the alignment with personal values [38]. This shift could explain the stronger presence of some altruistic motivations among older YAs in our study. Younger respondents in our study appeared to be more driven by some self-interest and personal motivations compared to older respondents. For instance, 50% of females <25 was motivated by learning about research, compared to 31% of those aged 25–29, 22% aged 30–34 and 23% aged 35–39. On the other hand, other personal motivations for participation, such as financial incentive, getting test results and study results, and self-interest, did not reveal significant differences between age groups. This finding suggests the importance of considering both direct personal gains and altruistic motivations to improve recruitment.

In terms of retention, the survey results indicated a strong preference for maintaining contact via email, which was viewed as the most reliable method by all participants. Based on input from a health research advisory board, email might also be viewed as the easiest method to discern if it is a legitimate communication, in contrast to texts or other methods. Social media also emerged as a popular tool for engagement, with platforms, such as Facebook and Instagram, being voted highly for their ability to keep participants informed and involved. The high demand for feedback on research outcomes (82%) suggests that participants value transparency and are more likely to remain engaged if they understand the impact of their contributions to research and the greater societal good. This finding highlights the potential of regular updates and results sharing as part of the retention strategy.

Our findings are consistent with prior research that emphasizes the importance of addressing both altruistic and personal benefits to retain participants in long-term studies [39]. In particular, altruistic motivations examined in our study included expressing the importance and benefits of research, interacting with local schools or community groups, sharing research with friends, and social value; while personal motivations included receiving study results, financial incentives, and personalized feedback. Fifty-eight percent of female YAs and 56% of male YAs ≥30 years indicated they would sustain study participation based on their perception of the importance and benefits of research, compared to 22% of female YAs and 32% male YAs <30 years old. Our study found that respondents considered providing regular updates and clear communication about the impact of the research, as well as offering tangible incentives, crucial in maintaining participant interest and engagement over time. Altruistic motivations can have a significant impact on initial engagement, while personal benefits might play a larger role in ensuring continued participation [37].

Our study identified social media platforms, particularly Facebook and Reddit, as effective channels for recruiting YAs into health studies. The effectiveness of Facebook recruitment was also endorsed in a prior study, which demonstrated its cost-efficiency in reaching youth with post-traumatic stress disorder (PTSD) for medical research [40]. Similarly, Docherty et al. (2019) also emphasize the growing preference for online recruitment, especially among AYA cancer survivors, who are more likely to interact with digital content than respond to in-person or mailed invitations [41]. Moreover, female participants from our study showed a stronger preference towards social media recruitment methods compared to males, which aligns with the Valle et al. study [42]. However, traditional methods, such as clinic-based recruitment, are still effective, especially when combined with online efforts [43]. Rabin et al. (2013) found that while online recruitment through social networks was effective, mailings also resulted in a substantial portion of participants, highlighting the importance of a mixed methods approach [43]. This dual approach is also supported in the study by Jaffee et al. (2009), which utilized community outreach and existing treatment program networks alongside media advertising to successfully recruit adolescents with co-occurring psychiatric and substance use disorders [28].

While studies examining retention strategies in YAs were more limited, our findings indicate that regular email communication, financial incentives, and sharing study results are endorsed as key to maintaining participation. This aligns with strategies highlighted in other studies. For example, Brownstone et al. (2012) reported high retention rates in an anorexia nervosa treatment trial by leveraging consistent follow-ups and strong involvement from medical providers [44]. Similarly, Bauermeister et al. found that financial incentives significantly boosted retention in a study on alcohol and drug use among YAs [45]. These studies emphasize the importance of structured communication and meaningful rewards, which is consistent with our approach. Moreover, Teague et al. (2018) highlighted that personalized follow-ups and flexibility in study protocols are effective in retaining participants [46], echoing our use of email as a primary tool for maintaining engagement. Additionally, Robinson et al. (2015) noted that sharing study results with participants enhances retention by making them feel valued [47], a strategy that also preferred in our study.

This study also has several limitations. Our study included only YAs aged 18–39 years due to the eligibility criteria of the MTurk platform, which requires participants to be 18 or older. As a result, we were not able to include adolescents under 18, a subgroup that is historically underrepresented in research [48]. Additionally, the participants were skewed toward aged 30 to 39 years, limiting the generalizability of our findings to the broader YA population. Including adults in the emerging age period of 18–24 years is essential to developing inclusive and effective strategies across the entire YA population. Our study was open to individuals in that age range; however, we were not as successful as we would have liked in recruiting younger adults. In addition, our participants lacked racial and ethnic diversity, with participants from non-Hispanic Black and Hispanic communities underrepresented in this study. This limitation reduces the applicability of our findings to these groups and points out the need for culturally sensitive and community-based recruitment and retention strategies. Research shows that adaptive outreach approaches, such as bilingual recruitment materials, engagement through trusted community organizations, and involvement of culturally tailored content, are important in improving participation among underrepresented racial and ethnic groups [49]. Future studies should prioritize inclusive recruitment approaches that reflect the demographic diversity of the YA population.

Another limitation is that the use of MTurk may have biased our sample towards individuals who favor online platforms. Respondents who are using MTurk are likely to represent a sample of those who are digitally comfortable and knowledgeable about the MTurk application and thus not fully representative of all individuals in this age range. For example, our participants had a higher education level than the general population [50,51]. The reliance on a digital platform like MTurk excludes those without regular access to digital devices or the internet, possibly skewing the data towards certain socioeconomic or demographic groups. However, the vast majority of YAs are “digital natives” and comfortable responding on the computer [35] and the utilization of the MTurk yielded a higher proportion of male respondents compared to other studies, which typically struggle to engage males during this developmental period [52,53]. Thus, we were able to tap into a demographic for whom much less is known about their motivations for joining a study and maintaining involvement. Our study demonstrated that online studies can be a successful route for engaging male respondents for researchers who are struggling to engage sufficient number of male participants in their studies. While our study provides valuable insights that could help tailor recruitment and retention strategies for digitally engaged YAs, future studies would benefit from integrating multiple recruitment methods, such as community outreach, clinical approaches, and social networks, to improve representativeness. Incorporating a qualitative component would allow deeper exploration of participants’ motivations, barriers, and experiences in research participation, and further advancing the understanding gained from survey-based findings. Lastly, our study did not differentiate between respondents based on specific health conditions or disease states; this limits our ability to understand how motivations for participation might vary among specific YA patient populations and should be the focus of future research.

While our study successfully identifies effective strategies for recruiting and retaining YAs, it also highlights several areas for further exploration. The rapid evolution of digital and social media presents both opportunities and challenges, requiring ongoing adaptation of strategies. Future research should investigate the development of dynamic social media outreach programs that can flexibly adapt to changing media consumption patterns and examine how these strategies perform across different YA subgroups, especially among younger adolescents. Moreover, incorporating insights from YA advisory boards will help create more engaging research environments that resonate with this demographic, further enhancing participation and retention, as these board members contribute to the development of patient-relevant care solutions [54]. By integrating feedback from these boards, researchers can develop studies that are not only more patient-centered, but also more likely to retain participation over time [55]. Advisory boards may also foster a trusted relationship between research and participants, which is important for maintaining long-term engagement in studies [56]. This is particularly applicable to YA research, as involving advisory boards can help address the unique challenges of this population. For instance, advisory boards can provide insights into effective communication strategies that resonate with YAs, thereby improving recruitment and retention rates. Future research could also explore the relationship between personal versus altruistic motivations and financial need. This exploration could provide a deeper understanding of how economic factors intersect with age-related motivations, offering more tailored strategies for engaging YAs in health research.

## Conclusion

This study highlights the need for research methodologies that align with the communication habits and preferences of YAs. By leveraging technology for enrollment and ensuring continuous engagement through transparent communication and feedback, researchers can enhance recruitment and retention rates while improving the quality of health research involving YAs. Our findings identify that YA participation is driven by a mix of altruistic motivations, such as contributing to the community and research, and personal motivations, including personal health benefits and financial incentives, emphasizing the need for strategies that address both aspects of recruitment and retention motivations. Innovative approaches to meet the evolving needs of the dynamic YA population can improve the representativeness of YAs in health research studies. Future efforts should also incorporate direct feedback from AYAs to ensure that research designs are effective and engaging.

## Supporting information

10.1017/cts.2025.10182.sm001 Wang et al. supplementary material Wang et al. supplementary material

## Supplementary material

The supplementary material for this article can be found at https://doi.org/10.1017/cts.2025.10182.
