# Supplementary material for: Recruitment and retention in young adult health research: Motivations and barriers
Source: J Clin Transl Sci. 2025 Oct 22;9(1):e250. doi: 10.1017/cts.2025.10182 (PMC12695509; doi:10.1017/cts.2025.10182)
Supplement: Wang et al. supplementary material [file S2059866125101829sup001.docx]

Supplemental Table 1. Selected Recruitment Survey Questions and Response Options.

| Survey Questions | Response Options |
| --- | --- |
| If you had the opportunity, would you consider participating in a health study or clinical trial? | Yes; no; maybe |
| Which of the following reasons would encourage you to take part in a health study (select all that apply): | Friend/family referral; Getting test results; Getting study results; Giving back to the community; Health care provider referral; Improving treatment, evaluation, or care for a health issue; Incentive (gift card, money, meal vouchers/food, giveaways, school credit); Learning about research; Self-interest; Time commitment of study; Other (please specify) |
| Would any of these reasons stop you from participating in a health study or clinical trial (select all that apply)? | Concern about sharing personal information; Concern or fear about possible side-effects; Concern that you will be in the control group and not get treatment; Concern or uncertainty about study procedures; Cultural barriers; Do not have the technology (computer, cellphone); Insufficient incentives; Lack of flexible scheduling; Language barriers; Limits your participation in another research study; Other time commitments (school, sports, job); Social pressure (stigma/worried about what others think); Takes too much time; Too expensive; Transportation challenges; Travel distance; Would need to miss work; Other (please specify) |
| Would your interest in joining a study differ depending on whether the study was online or in person? | Yes; no; maybe |
| Have you heard about opportunities to participate in health studies or clinical trials in the past? | Yes; no; maybe |
| Please select any of the social media platforms where you have heard about health studies or clinical trials in the past (select all that apply): | Discord; Facebook; Instagram; LinkedIn; Pinterest; Reddit; Snapchat; TikTok; Twitch; Twitter; WhatsApp; YouTube; None; Other |
| Please select any of the other ways that you have heard about health studies or clinical trials in the past (select all that apply): | Billboards; Community Events; E-mail; Family; Flyers; Friend/acquaintance; Healthcare provider; Letter or brochure in the mail; Newspaper advertisement; Online website that lists studies that are recruiting participants (such as Study Pages); Phone calls; Radio; School announcements or events; Television; Text message; None; Other |
| What do you think are the best ways to send out information about health studies to people your age (select all that apply)? | Advertisement at community events and spaces (such as businesses or churches); Billboards; Community Events; E-mail; Family; Flyers; Friend/acquaintance; Healthcare provider; Letter or brochure in the mail; Newspaper advertisement; Online website that lists studies that are recruiting participants (such as Study Pages); Phone calls; Radio; School announcements or events; Social Media (list types); Television; Text message; Other |
| What is the best way to motivate people in your age group to participate in research studies (select all that apply)? | Academic credit; Certificate to show participation in the study; Express the importance and benefits of research; Gift Cards; Interact with local schools or community groups, including businesses and churches; Make part of a club; Meal Vouchers; Money; Self-interest/topic interest; Share research with friends; Share the study results with participants; Social media shout-out to participants; Social value (people see that you are part of the study); T-shirts, water bottles, or other free “swag” items; Volunteer credits; Other |

Supplemental Table 2. Selected Retention Survey Questions and Response Options.

| Survey Questions | Response Options |
| --- | --- |
| What do you think is the best way to keep in contact with people your age over a period of multiple weeks, months, or years (select all that apply)? | Emails; Flyers or letters sent in the mail; Meetings; Newsletter; Telephone calls; Text messages; Social media; Other |
| Please select any of the following social media platforms that you think would be best to keep in contact with participants (select all that apply): | Discord; Facebook; Instagram; LinkedIn; Pinterest; Reddit; Snapchat; TikTok; Twitch; Twitter; WhatsApp; YouTube; None; Other |
| What do you think would be the best way(s) to keep health study participants that are your age interested and involved in a long study (select all that apply)? | Foster relationships; Check-in; Birthday cards; Memes; Text messages; Provide information on study results; Invite the participant to meet-up; Form a community with other participants; Other |
| What do you think is the best way to motivate people in your age group to continue to participate in a health study or clinical trial over an extended period of time? Select all that apply. | Academic credit; Certificate to show participation in the study; Express the importance and benefits of research; Gift Cards; Interact with local schools or community groups, including businesses and religious organizations; Make part of a club; Meal Vouchers; Money; Self-interest/topic interest; Share research with friends; Share their results with participants (results from tests, scans or treatment); Share the study results with participants; Social media shout-out to participants; Social value (people see that you are part of the study); T-shirts, water bottles, or other free “swag” items; Volunteer credits; Other |
| If you participated in a health research study or clinical trial, would you want to learn about the results of the research? | Yes; No; Not Sure |
| If you participated in a health research study or clinical trial, what do you think would be the best way to share the research results with you (select all that apply)? | Blogs; Email; Meeting with researcher; Newsletters; Podcast; Post the studies on a website; Publish the results in a paper; Social media; Texts; Videos; Other |
| Please select any of the following social media platforms that you think would be best to share the research results with participants (select all that apply): | Discord; Facebook; Instagram; LinkedIn; Pinterest; Reddit; Snapchat; TikTok; Twitch; Twitter; WhatsApp; YouTube; None; Other |
